# Decolorization and detoxification of Synozol red HF-6BN azo dye, by *Aspergillus niger* and *Nigrospora* sp

**DOI:** 10.1186/1735-2746-10-12

**Published:** 2013-01-21

**Authors:** Sidra Ilyas, Abdul Rehman

**Affiliations:** 1 Department of Microbiology and Molecular Genetics, University of the Punjab, Lahore, Pakistan

**Keywords:** Azo dyes, Decolorization, *Aspergillus niger*, *Nigrospora* sp, Bioremediation

## Abstract

In the present investigation the fungi, *Aspergillus niger* and *Nigrospora* sp. were employed for decolorization of Synozol red HF-6BN. Decolorization study showed that *Aspergillus niger* and *Nigrospora* sp. were able to decolorize 88% and 96% Synozol red 6BN, respectively, in 24 days. It was also studied that 86% and 90% Synozol red containing of dye effluent was decolorized by *Aspergillus niger* and *Nigrospora* sp. after 28 days of incubation at room temperature. A fungal-based protein with relative molecular mass of 70 kDa was partially purified and examined for enzymatic characteristics. The enzyme exhibited highest activity at temperature ranging from 40-50°C and at pH=6.0. The enzyme activity was enhanced in the presence of metal cations. High performance liquid chromatography analysis confirmed that these fungal strains are capable to degrade Synozol red dye into metabolites. No zones of inhibition on agar plates and growth of *Vigna radiata* in the presence of dye extracted sample, indicated that the fungal degraded dye metabolites are nontoxic to beneficial micro-flora and plant growth. *Aspergillus niger* and *Nigrospora* sp. have promising potential in color removal from textile wastewater-containing azo dyes.

## Background

Synthetic dyes are extensively used in textile dyeing, paper printing, color photography, pharmaceutical, food, cosmetics and other industries. During textile dyeing, the amount of dye lost in the effluents is dependent upon the class of dye used, varying from only 2% loss when using basic dyes to a 50% loss when reactive dyes are used [1]. Approximately 20% of the losses enter the environment through effluents from wastewater treatment plants [2].

Azo compounds are a broad class of organic compounds with formula R-N=N-R^′^, in which R and R^′^ can be either aryl or alkyl groups. Azo compounds are solids of varying color from yellow to red and violet to blue. The N=N group is called an azo group and parent compound, HNNH, is called diimide. Synozol Red HF-6BN, an azo reactive dye, is a representative of a dye class to be recalcitrant with a conventional wastewater treatment system. Reactive dyes are easily soluble in water, therefore they have little affinity to be adsorbed on biomass and generally pass through activated sludge systems [3].

It is quite undesirable to discharge azo dyes with different colors into the environment due to their color pollution, biorecalcitrance and toxic intermediates, since the cleavage of azo bonds produces aromatic amines which are considered mutagenic and carcinogenic [4,5]. In addition, some azo dyes or their metabolites because of low biodegradability may be mutagens or carcinogens to humans as well as to other animals [5,6]. Therefore, considerable attention has been given to evaluating the fate of azo dyes during wastewater treatment and in the natural environment. The effluents of these industries are highly colored and disposal of these wastes into natural waters causes damage to the environment [7,8].

Decolorization of these dyes by physical or chemical methods including flotation, hyper filtration, [9], adsorption, coagulation-flocculation, ion-exchange, oxidation, electrochemical methods [10,11] and precipitation methods, chemical degradation or photodegradation is financially and often also methodologically demanding, time-consuming and mostly not very effective [12]. The above mentioned ways for clean-up are expensive, coupled with the formation of large amount of sludge and the emission of toxic substances [13], which limit their application [14].

Compared with chemical and physical methods, a number of studies have focused on biological treatment, in which microorganisms which are able to decolorize and biodegrade these azo dyes are used, thus producing lower costs and fewer toxic resultants [15]. Several combined anaerobic and aerobic microbial treatments have been suggested to enhance the degradation of azo dyes [5]. Alternatively, dye decolorization using microbial enzymes has received great attention in recent years due to its efficient application [16-18].

Color removal processes with active microorganisms have two different simultaneous steps: adsorption of dyes on the surface of the organisms and degradation of dyes by the enzymes produced by these organisms [19-21]. Decolorization of textile dye effluent does not occur when treated aerobically by municipal sewage systems [22]. Brightly colored, water-soluble reactive and acid dyes are the most problematic, as they tend to pass through conventional treatment systems unaffected [22]. The possibility of using fungi to decolorize wastewater containing dyes has received much attention because their ligninolytic enzymes have the ability to degrade many recalcitrant pollutants, including synthetic dyes. Biocatalytic processes based on fungi provide alternative methods to decolorize textile effluents [23-25].

The aim of this work was to exploit the biodecolorization of Synozol red HF-6BN by fungi with the following objectives: (1) to assess the ability of the fungal cultures to decolorize the actual dye industry waste, (2) to confirm the degradation of the dye, and (3) to assess the toxicity of the degraded products.

## Materials and methods

### Microorganisms and growth conditions

The fungi, *Aspergillus niger* and *Nigrospora* sp. were a kind gift from Dr. Arshad Javaid, Institute of Plant Pathology, Punjab University, Lahore, Pakistan. The solid medium used for fungal growth contained per liter: 10 g of malt extract, 4 g of yeast extract, 4 g of glucose and 20 g of agar (pH=5.5). For laccase production and induction studies, 3 mL of homogenized mycelium were used for inoculation of 1000-mL Erlenmeyer flask containing 300 mL of culture medium. This salt basal medium contained (per liter) glucose, 10 g; peptone, 5 g; yeast extract, 1 g; ammonium tartrate, 2 g; KH_2_ PO_4_, 1 g; MgSO_4_.7H_2_O, 0.5 g; trace elements solution, 1 mL. The pH was adjusted to 5.5.

### Decolorization study

Dye concentration of the decolorized broth was quantified by comparing its absorbance with the absorbance of known concentrations of Synozol red HF-6BN and this was used to calculate the dye removal rate (mg L^-1^) and expressed in percentage of decolorization [26].

(1) $$\mathrm{Decolourization}\;\left(\%\right)=\frac{I-F\;X\;100}{\text{I}}$$

Where I = initial absorbance and F = absorbance of decolorized medium.

### Decolorization of dye manufacturing industry’s effluent

To check the efficacy of fungi to decolorize the industrial effluent a lab-scale experiment was set up. Three plastic containers were taken. In the first two containers 8 L of dye effluent was taken along with 1.5 L of *A. niger* and *Nigrospora* sp. grown cultures in basal salt medium. In the third container only 8 L of dye effluent (temperature=33°C; pH=7.8; dissolved oxygen= 0.146±0.04 g/L; Synazol=2.140±0.03 μg/mL) was taken and 20 mg/L of Synazol red stress was maintained in each container. Experiment was carried out at room temperature (28±2°C). After 14 and 28 days of incubation samples were taken, centrifuged and supernatants were used to estimate the amount of Synazol in dye effluents by UV–vis spectroscopic analysis (Hitachi U-2800, Tokyo, Japan). The decolorization percent was calculated by taking untreated dye solution as control (100%).

### Partial purification and SDS-PAGE analysis of laccase

The fungal cultures supernatants were harvested by centrifugation at 6000 × g for 10 min at 4°C and concentrated by addition of solid ammonium sulfate (60%). The precipitate was harvested by centrifuging at 6000 × g for 10 min and dialyzed (Cellu·Sep membrane cat no 5-5050-34; pore size 34 mm for mw of protein 50,000) against 10 mM sodium tartrate buffer (pH=5.5) for overnight. The dialyzed sample was centrifuged at 6000 × g for 1.5 h in centricon Ultracel YM-100 membrane (100,000 NMWL) just to remove and concentrate the protein having molecular weight lower than 100 kDa. The protein solution above the centricon was discarded and flow through was taken, having proteins less than 100 kDa. This protein solution was further used in Ultracel YM-30 membrane (30,000 NMWL) to remove protein having MW 30 or less than 30 kDa. The above protein solution was further used in Ultracel YM-50 membrane (50,000 NMWL) to remove the protein with MW 50 kDa. The protein solution in upper chamber of the centricon was further concentrated in concentrator (Eppendorf concentrator, 5301) at 4°C and was finally analyzed by sodium dodecyl sulfate (SDS)-Polyacrylamide gel electrophoresis (PAGE) as described by Laemmli [27]. The protein concentration was determined by Bradford assay using bovine serum albumin (BSA) as a standard.

### High-performance liquid chromatography (HPLC)

The biodegraded products were monitored by HPLC. HPLC analysis was carried out (Waters model no. 2690) on a C18 column (symmetry, 250 × 4.6 mm) with methanol: acetonitrile (1:1) as mobile phase with at flow rate of 1.0 mL/min and UV detector at 540 nm [28].

### Enzyme assay

Laccase activity was determined by using ABTS [azino-obis (3-ethylbenzothiazoline-6-sulfonic acid) Sigma, USA] as substrate at 465 nm. The measurements were made with 100 mM sodium acetate buffer (pH=5.0) at 30°C for 30 min. One unit of enzyme activity was defined as a change in absorbance U/mL/min of enzyme. The relative percentage activity was calculated by the change in absorbance in reaction mixture without enzyme and with enzyme.

### Effect of temperature, pH and metal ions on the enzyme activity

The optimum temperature of the partially purified laccase was determined by incubating the reaction mixture for 30 min at different temperatures ranging from 30°C to 90°C. The pH profile of the enzyme was evaluated by incubating the reaction mixture for 30 min in the presence of appropriate buffers: 50 mM sodium acetate (pH=4.5-6.0), 50 mM sodium phosphate (pH=6.0-8.0), and 50 mM Tris–HCl (pH=8.0-10.0). The activity of each sample was quantified by the assay method as described above.

The metal ion effect on enzyme activity was examined by chloride salts of various metals. Each metal was added in the reaction mixture at a final concentration of 0.1 mM and laccase activity was determined at 30°C and pH=5.0. No metal ion was added in the control assay.

### Microbial and phytotoxicity toxicity

The decolorized dye at the concentration of 100 mg/L was tested for its toxic effect [29] on the agriculturally important soil bacterial flora according to [30]. *Bacillus cereus* and *Azotobacter* sp. were inoculated on minimal salt medium. Two wells of 2 mm diameter were made on the minimal salt medium containing plates. Both were filled with 1.0 mL of decolorized centrifuged broth. The plates were incubated at 30°C for 48 h. Zone of inhibition surrounding the well represented the index of toxicity.

Phytotoxicity test was also performed in order to assess the toxicity of the treated dye sample by fungi at concentration of 100 mg/L according to [28]. For this purpose soil was sterilized by autoclaving and almost equal quantity of sterilized soil was taken in pots. Seeds of mung beans (*Vigna radiata*) were taken and sterilized with HgCl_2_ (1%). After washing thrice with HgCl_2_, the beans were rinsed thoroughly with distilled water and were inoculated (four in each pot). Pots were watered regularly with 15 mL of supernatant of 7 days old cultures of *A. niger* and *Nigrospora* sp. For control pots tap water was used instead of fungal supernatant. Growth of *Vigna radiata* watered with fungal decolorized water was compared with the growth of *Vigna radiata* watered with simple tap water after 7 days incubation of 12 (dark): 12 (light) time periods.

### Statistical analysis

Observations were made and all the experiments were repeated two or more times and the results reported are average values.

## Results

### Decolorizing ability of the fungi

Synozol Red processing capability of the fungal strains was checked by adding dye at 20 mg/L in the culture medium. *A. niger* could decolorize 88% of dye from the medium after 24 days of incubation. The fungus was also capable to decolorize dye by 34%, 56% and 78% from the medium after 6, 12 and 18 days, respectively (Table 1). *Nigrospora* sp. could decolorize 96% of dye from the medium after 24 days of incubation. *Nigrospora* sp. was also capable to decolorize dye from the medium after 6 (35%), 12 (60%) and 18 (82%) days (Table 1).

**Table 1:** Decolourization (%) of Synozol red HF-6BN by fungal isolates incubated at 30°C for different time period

**Fungal isolates**	**6 d**	**12 d**	**18 d**	**24 d**
*A. niger*	34	56	78	88
*Nigrospora* sp.	35	60	82	96

### Decolorization of Synozol Red from industrial effluent

**Figure 1:**
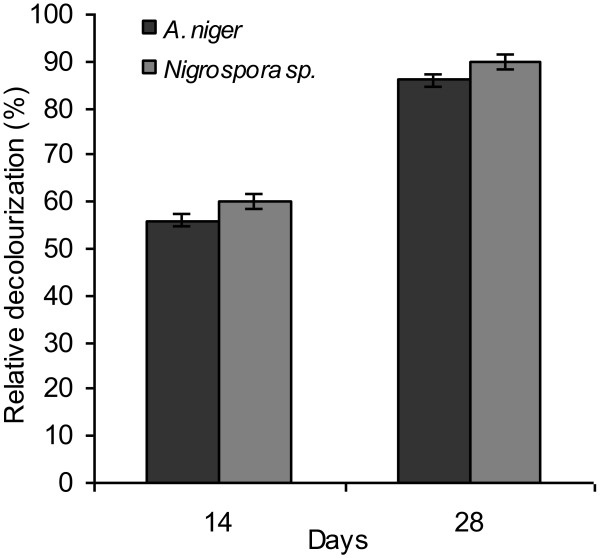
Decolorization of Synazol red HF-6BN by fungi from the industrial effluent after 14 and 28 days of incubation at room temperature.

In order to assess the ability of fungi to remove Synozol Red from dye contaminated industrial effluents, a lab-scale experiment was performed. *A. niger* was capable to decolorize 60% and 86% dye from the industrial effluent after 14 and 28 days of incubation at room temperature. Similarly 60% and 90% dye-containing industrial effluent was decolorized after 14 and 28 days of incubation at room temperature by *Nigrospora* sp. (Figure 1).

### Effect of temperature on enzyme activity

**Figure 2:**
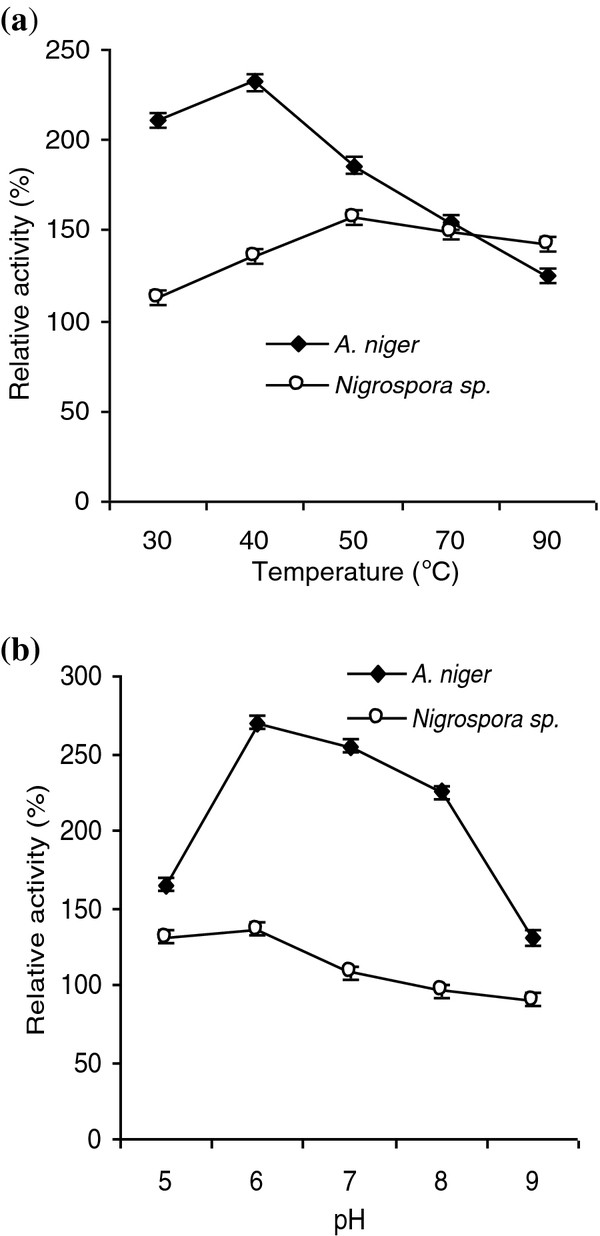
**Effect of temperature and pH on the enzyme activity of laccase.** (**a**) Effect of temperature on laccase activity. The enzyme activity was measured at various temperatures at pH=5.0 without addition of any metal ions in the reaction mixture. (**b**) Effect of pH on laccase activity. Activity was examined as described in the experimental section.

Extracellular laccase produced by *A. niger* and *Nigrospora* sp. was characterized with regard to pH optimum and thermostability. The activity of crude laccase isolated from culture filtrate of *A. niger* and *Nigrospora* sp. was determined at various pH values and temperatures. *A. niger* laccas activity was maximum at 40°C (232%) whereas at 30°C (211%), 50°C (186%) and 70°C (155%) 90°C (125%) at pH=6 were also determined. Enzymatic activity of *Nigrospora* sp. was best at 50°C (152%) whereas at 30^0^C (113%), 40°C (136%), 70°C (149%) and 90°C (142%) were also estimated (Figure 2a).

### Effect of pH on enzyme activity

Experiments were performed to elucidate whether pH interfere laccase activity or not and it was assessed that *A. niger* laccase activity was maximum at pH=6 (270%), in contrast to pH=5 (165%), pH=7 (255%), pH=8 (225%), and pH=9 (130%) showed decrease in enzymatic activity. *Nigrospora* sp. showed maximum activity at pH=6 (136%) whereas at pH=5 (131%), pH=7 (108%), pH=8 (96%) and pH=9 (90%) were observed (Figure 2b).

### Effect of metal ions on enzyme activity

**Figure 3:**
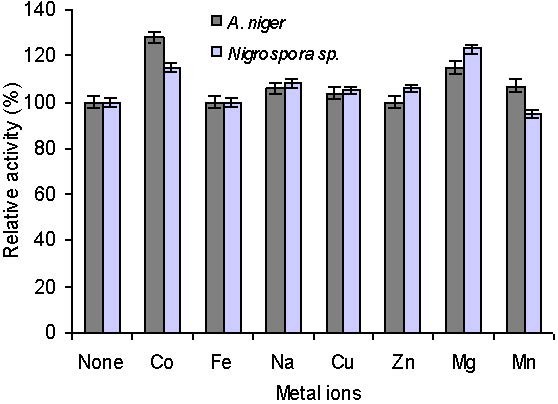
**Effect of metal ions on the enzyme activity of laccase.** A chloride salt of each metal cation was used at a final concentration of 0.1 mM. The activity was examined at 30°C and pH=5.0 as described in the experimental section.

Laccase activity of *A. niger* was enhanced by 28%, 7%, 6%, 4% and 15% in Co^2+^, Mn^2+^, Na^+^, Cu^2+^ and Mg^2+^ and there was no change in enzymatic activity in Fe^2+^ and Zn^2+^. Enhanced laccase activity in the presence of Co^2+^ (15%), Na^+^ (8%), Cu^2+^ (5%), Zn^2+^ (6%) and Mg^2+^ (23%) were also observed in the laccase isolated from *Nigrospora* sp. The 5% decrease in the activity of enzyme in the presence of Mn^2+^ was observed (Figure 3). It is clear from the laccase activity that the activity was not dependent on the divalent metal ions.

### Laccase purification

**Figure 4:**
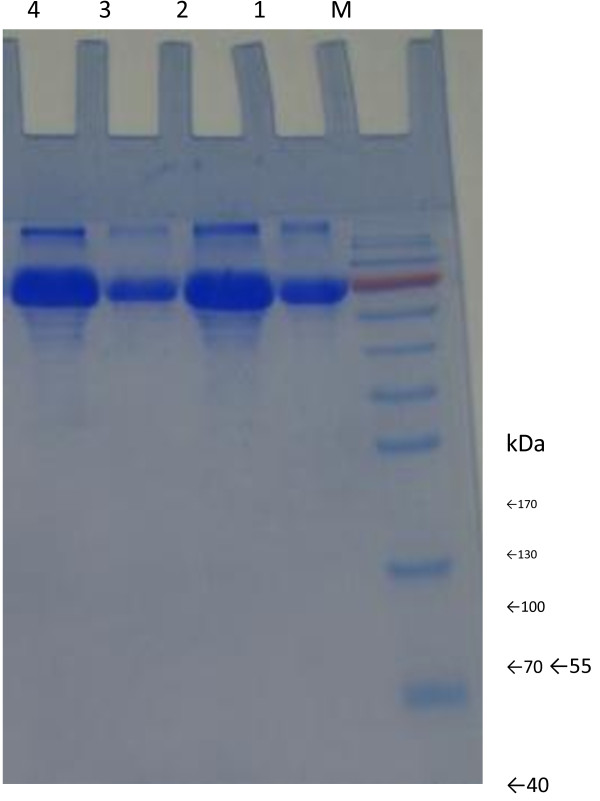
**Coomassie brilliant blue-stained SDS-PAGE of partially purified laccase from *A. niger *and *Nigrospora *sp.** Lane M, molecular mass protein makers; lanes 1–3, ammonium sulfate precipitated samples after dialysis against 10 mM sodium tartrate buffer; lanes 2–4, eluate after Ultracel YM membranes.

Sodium dodecyl sulphate-polyacrylamide gel electrophoresis (SDS-PAGE) was used to determine the relative molecular weight of the partially purified enzyme. The partially purified protein band corresponds to laccase with a molecular weight of about 70 kDa in *A. niger* and *Nigrospora* sp*.* was visualised by Coomassie brilliant blue staining (Figure 4). These results suggest that enzyme from both fungal strains is monomeric protein.

### HPLC analysis

**Figure 5:**
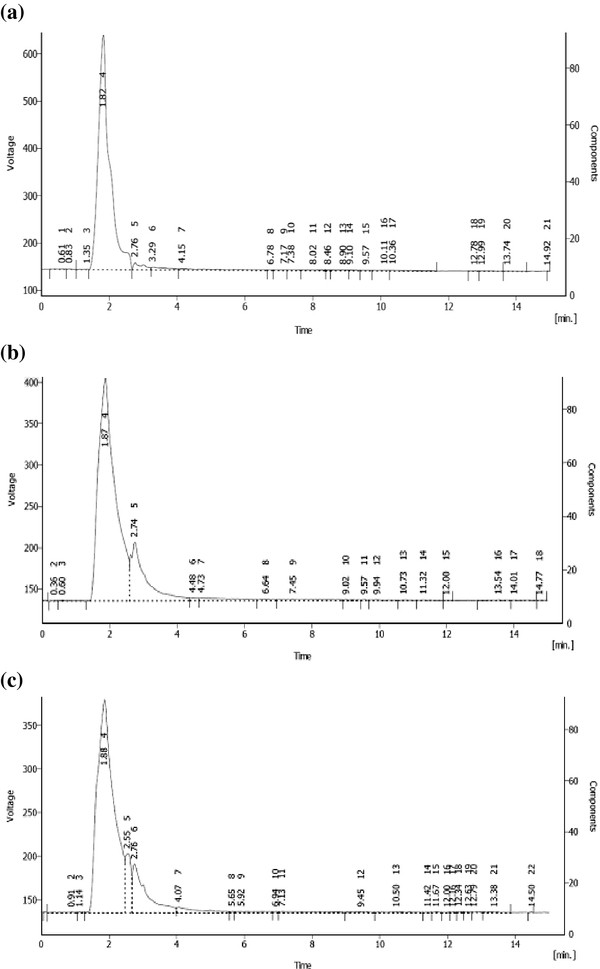
**HPLC analysis of reactive Synazol red HF-6BN with a mobile phase of profile of methanol: acetonitrile (1:1) at 1.0 mL/min.** The column was C-18 (250×4.6 mm). (**a**) represents dye chromatogram while (**b**) and (**c**) represent *A.niger* and *Nigrospora* sp. degraded dye products extracted after 7 days of incubation at 30°C.

The HPLC analysis of the dye sample collected at 0 h incubation showed three detectable peaks at 1.82, 2.76 and 3.29 min (Figure 5a). An extracted sample (7 days) showed two detectable peaks at retention times of 1.87 and 2.74 min. The third peak visible in the control has not been visible in *A. niger* treated dye sample (Figure 5b). Similarly in case of *Nigrospora* sp. degraded dye extracted sample showed three detectable peaks at different retention times of 1.88, 2.55 and 2.76 min (Figure 5c) indicating biodegradation.

### Toxicity assay

**Figure 6:**
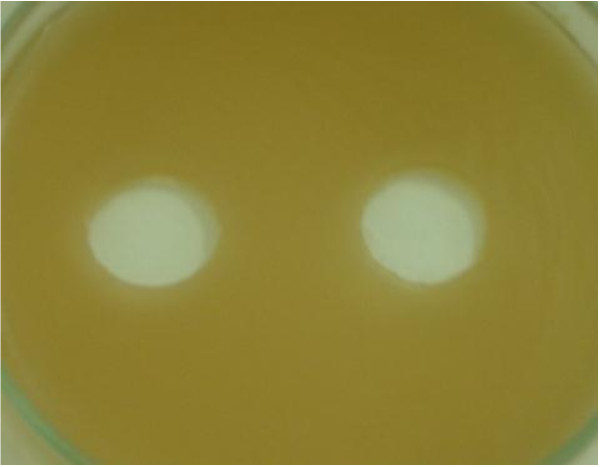
**No zone of inhibition was observed around the fungal-decolorized broth after incubating *B. cereus *at 30°C for 48 hours.**

**Figure 7:**
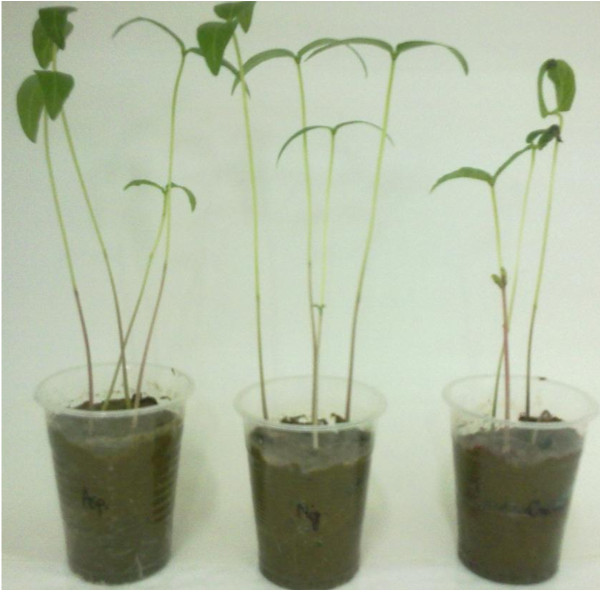
**The growth of *Vigna radiata *in dye effluent (right) and fungal treated [left (*A. niger*) and middle (*Nigrospora *sp.)] wastewater indicates that this fungal-treated wastewater is safe for plant growth.**

No zone of inhibition observed in the treated dye, indicated that the biodegraded or decolorized product was nontoxic to the tested beneficial bacterial flora of the soil (Figure 6). In the present investigation phytotoxicity study showed good germination rate of *Vagina radiata* in the fungal decolorized water and in tap water indicating that metabolites of the dye produced in the presence of fungi are found to be nontoxic to the growth of the plants (Figure 7).

## Discussion

Biodecolorization of dyeing wastewaters by microbial enzymes is a promising, eco-friendly and cost competitive alternative. Usman et al. [31] described that *Corynebacterium* sp. could decolorize 60% (Reactive black5) and 76% (Reactive yellow15) from the medium containing 100 mg/L after 2 days. Degradation of azo dyes by filamentous fungi, such as white rot fungi have already been reported [32]. Comapred to other fungal oxidative enzymes, laccases can act oxidatively, non-specifically at the aromatic rings and have the potential to degrade a wide range of compounds. Laccases have gained much attention over the last number of years in many industrial and environmental fields due to their wide substrate specificity [33,34].

Ali *et al.*[35] reported that the decolorization of Acid red 151, Orange II, Sulfur black and Drimarine blue K_2_RL were 68.64%, 43.23%, 21.74%, and 39.45%, respectively by *A. niger* in liquid medium under static condition. Jin et al. [36]) reported 89.9% optimum decolorization rate of Reactive black RC, Reactive yellow HF2-GL, Reactive blue BGFN and Reactive black B-150 at pH=3.0 after 48 h of incubation. Similarly removal of Congo red from an aqueous solution by fungus *A. niger* was reported by Fu and Viraraghavan [37]. Zahmatkesh *et al.*[38] reported that 70±3% decolorization of Reactive Orange 16 was achieved after 6 hours of dye addition by *Phanerochaete chrysosporium* fungus immobilized in calcium alginate biogel beads**.** In the present investigation *A. niger* and *Nigrospora* sp. could decolorize 88% and 96% of dye from the medium after 24 days of incubation at 28±2°C.

Laccases from basidiomycetes are generally monomeric protein with a molecular mass between 50 and 80 kDa [39]. The two laccases purified from *Trametes trogii* are monomeric proteins with the same molecular mass (62 kDa). Garzillo *et al.*[40] reported a 70 kDa laccase from *Trametes trogii* strain 201 while Coll *et al.*[41] reported 64 kDa laccase from Basidiomycete PM1. A 97 kDa denatured laccase by SDS-PAGE analysis was reported by Han *et al.*[42]. In the current study a 70 kDa laccase, monomeric protein, has been partially purified from *A. niger* and *Nigrospora* sp*.* (Figure 4).

The optimum temperature of the laccase was 50°C with ABTS as a substrate in buffer of pH=5. Optimum temperature of laccase I from *Pleurotus eryngii* was 65°C and that of laccase II from the same organism was 55°C [43]. Zouari-Mechichi *et al.*[44] reported that *T. trogii* laccase in crude form showed optimum activity at pH=7 at room temperature for 24 h but retained more than 50% of its activity at pH =5. The laccase in the crude extract was also stable for 24 h at 50°C. In case of *A. niger* the maximum laccase activity was estimated at 40°C (232%) whereas in *Nigrospora* sp. the maximum enzyme activity was determined at 50°C (152%) during the present investigation (Figure 2a).

Jung *et al.*[45] reported that laccase of *Trichophyton rubrum* was more stable at pH=6, although pH optima depend on the substrate used [46]. The activity of many laccases decreases beyond optimum pH [45], but this laccase showed a high relative activity over a broad pH range from 5 to 9. This could be a very useful characteristic for various industrial applications. The maximum relative increase in laccase activity was 28% (Co^2+^) and 23% (Mg^2+^) in *A. niger* and *Nigrospora* sp., respectively. No change in laccase activity was estimated in the presence of Fe^2+^ and Zn^2+^ in *A. niger* while 5% activity decrease was determined in the presence of Mn^2+^ in *Nigrospora* sp. (Figure 3).

Brilliant green, Fast green, Methylene blue, and Congo red removal and their toxicity after biological treatment have been reported by [30]. Despite the fact, untreated dyeing effluents may cause the serious environmental and health hazards. These effluents are being disposed off in water bodies and this water can be used for the agriculture purpose. In the present study phytotoxicity study showed good germination rate of *Vagina radiata* in the metabolites extracted after decolorization and in dye untreated wastewater indicating that metabolites of the dye produced are found to be nontoxic to the growth of the plants (Figure 7). Dawkar *et al.*[28] reported that metabolites were nontoxic with respect to *Sorghum bicolor* and *Triticum aestivum*. Similarly [47] reported that *Sorghum vulgare* and *Phaseolus mungo* showed good germination rate as well as significant growth in the plumule and radical for both the plants, in the Red BLI metabolites extracted after decolorization, as compared to dye sample.

Spadaro and Renganathan [48] concluded that the azo dyes are cleaved by fungal enzymes with the formation of quinonoe and diazene derivatives and release of the azo linkage as molecular nitrogen. Therefore, the formation of aromatic amines, which are suspected to be carcinogenic, would be prevented. Another advantage of fungal use is that the fungal transformations are mediated by exoenzymes [49] and therefore the rate-limiting membrane permeation of the substrate is not required. In contrast to fungal transformations, uptake of azo dyes into the cell is necessity for bacterial degradation.

## Competing interests

We (both authors) declare that we have no competing interests.

## Authors’ contributions

S. Ilyas carried out the decolorization, enzyme assays and microbial toxicity studies and A. Rehman participated in partial purification of laccase from the fungal culture and drafted the manuscript.
